# State of Oral Mucosa as an Additional Symptom in the Course of Primary Amyloidosis and Multiple Myeloma Disease

**DOI:** 10.1155/2014/293063

**Published:** 2014-06-09

**Authors:** Maciej R. Czerniuk, Artur Jurczyszyn, Grzegorz Charlinski

**Affiliations:** ^1^Department of Oral Medicine and Periodontal Diseases, Medical University of Warsaw, ul. Miodowa 18, 00-246 Warsaw, Poland; ^2^Department of Hematology, University Hospital in Cracow, ul. Kopernika 17, 31-501 Cracow, Poland; ^3^Department and Clinic of Haematology, Oncology and Internal Medicine, Medical University of Warsaw, ul. Banach 1A, 02-097 Warsaw, Poland

## Abstract

Multiple myeloma (myeloma multiplex (MM)) is a malignant non-Hodgkin's lymphoma derived from B cell. Its essence is a malignant clone of plasma cells synthesizing growth of monoclonal immunoglobulin, which infiltrate the bone marrow, destroy the bone structure, and prevent the proper production of blood cells components. The paper presents a case of 62-year-old patient who developed symptoms in addition to neurological and haematological changes in the oral mucosa in the course of multiple myeloma. The treatment resulted in partial improvement. The authors wish to draw attention not only to nonspecificity and rarity of changes in the mouth which can meet the dentist but also to the complexity of the multidisciplinary therapy patients diagnosed with MM.

## 1. Introduction 

For the last twenty years, an increasing number of patients have been referred to specialised dental offices with alarming symptoms and pathological changes in their mouth [1–5]. The latter concerns not only the marginal tissue (tooth root, periodontium, bone of alveolar process, and gingival margin) and/or apical periodontium (root apex and gingiva proper) but also lips, cheek mucosa, hard palate, soft palate, palatine arches, tongue, and sublingual region of oral cavity.

Multiple myeloma (MM) is one of the most common haematological tumours. In Poland, approximately 1500 new cases of MM are diagnosed every year. MM develops in 4 to 5 per 100 000 people per year and frequency increases with age. The median age of diagnosis is between 60 and 70 years of age. It is a malignant tumour classified by WHO as a non-Hodgkin lymphoma derived from B lymphocytes. It consists in the growth of a plasma cells clone synthesizing monoclonal immunoglobulins or their fragments that infiltrate the bone marrow thus destroying the bone structure, also in facial parts of the skull and in periodontium. This prevents the creation of appropriate blood morphotic elements. Patients very often suffer from progression of periodontal disease (PD) which, in the beginning, leads to gum bleeding and later to pathological loosening of teeth, atrophy of bone support and of surrounding periodontal tissues, and eventually tooth loss. These changes are accompanied by other pathological symptoms in the mucosa and especially in the tongue [6–9].

The medicines from the group of bisphosphonates are used in the treatment of MM. They can cause osteonecrosis of the jaw (ONJ) [10, 11]. In 2003 the first case of ONJ was described in a patient who was treated with bisphosphonates—BRON (bisphosphonate-related to osteonecrosis). The American Association of Oral Maxillofacial Surgeons (AAOMS) defined three pathognomonic criteria on which BRON relies:

1. the patient must be taking or have taken bisphosphonate medication;
2. the patient possesses an area of exposed bone in the jaw persisting for more than 8 weeks;
3. the patient must present no history of radiation therapy to the head and neck.

The majority of BRON clinical cases were linked with intravenous administration of bisphosphonates. There were also some cases of BRON after oral supplementation; however they were very rare. It seems that an important factor of this complication is a previous injury in the oral cavity (postextraction wounds). Nevertheless there were also some cases of spontaneous occurrence not related to any intra- or postoperative injuries. Except for BRON symptoms other conditions may cooccur in the maxilla and mandibular active suppurative fistulas, inflamed granulation tissue, and local superinfections, which require long-term treatment with antibiotics [9, 12, 13].

Amyloidosis is a condition that shows no symptoms for a very long time and it is diagnosed by finding amyloids in the biopsy specimen of oral mucosa and/or skin. In many cases amyloidosis can be defined as a disease where the general condition is getting worse while there are no obvious causes. Clinically it manifests itself by renal or heart failure, polyneuropathy, carpal tunnel syndrome, enlarged liver, malabsorption of unknown cause, or orthostatic hypotension. A characteristic clinical symptom is an enlarged tongue in the mouth. Amyloidosis treatment depends on how advanced the disease is when diagnosed and also on the patient's age and concurrent conditions. The major decision to be made is whether the patient can be qualified for a bone marrow transplant or not [9, 14].

In this study the authors want to present the case of a patient treated for multiple myeloma MM (plasma cell myeloma) and for primary amyloidosis AMD with complications: chest pain and exophytic changes in oral mucosa that occurred during the treatment. The cardiological aspects were previously described in a different medical review [15]; thus this study will only present the aspect of oral mucosa and periodontium diseases.

## 2. Case Report 

A 62-year-old patient reported to the Department of Oral Medicine and Periodontal Diseases, Medical University of Warsaw, for consultation and treatment of changes on the surface of oral mucosa.

The patient underwent left wrist surgery for carpal tunnel syndrome in 2001. He had an open, multiple fracture of distal tibia—skiing injury and in 2002 Th10 vertebra fracture with compression of the spinal cord and in 2007 decompression for spinal canal stenosis at L3–L5 by fenestration—which could have been the first symptom of MM. Moreover the patient underwent the diagnosis of muscle sclerosis and was suspected to have myotonic dystrophy type 2.

The biopsy from the lower lip showed hyaline degeneration and fibrosis as well as focuses of Langhans giant cells. This allowed excluding the presence of neoplastic metaplasia pattern.

In 2007 the patient underwent a surgery for lumbar spinal canal stenosis at L3-L4 and L4-L5 as he showed symptoms of neurogenic claudication. However the treatment did not bring about any improvement. In July 2008 the patient stayed at the Neurology Clinic of the Medical University of Warsaw because of muscle rigidity that has been progressing for 2 years. The conducted examination indicated the presence of monoclonal protein (Bence-Jones protein) in the serum and the urine as well as high concentration of *β*2-microglobulin in trepanobiopsy. MM was diagnosed. The patient was later hospitalized in the Haematology Clinic of the Medical University of Warsaw, where he underwent two autologous bone marrow transplants after induction therapy.

During the dental examination in question the patient said that he regularly went to see a dentist—average twice a year. These visits covered comprehensive dentistry (treatment of dental caries), endodontics (root canal therapy), dental surgery (teeth extraction), periodontology (scaling and root planning), and prosthetic dentistry (fixed denture, maxilla crowns; mobile denture, mandibular framework). The patient had two panoramic radiographs from 2006 and 2008.

**Figure 1:**
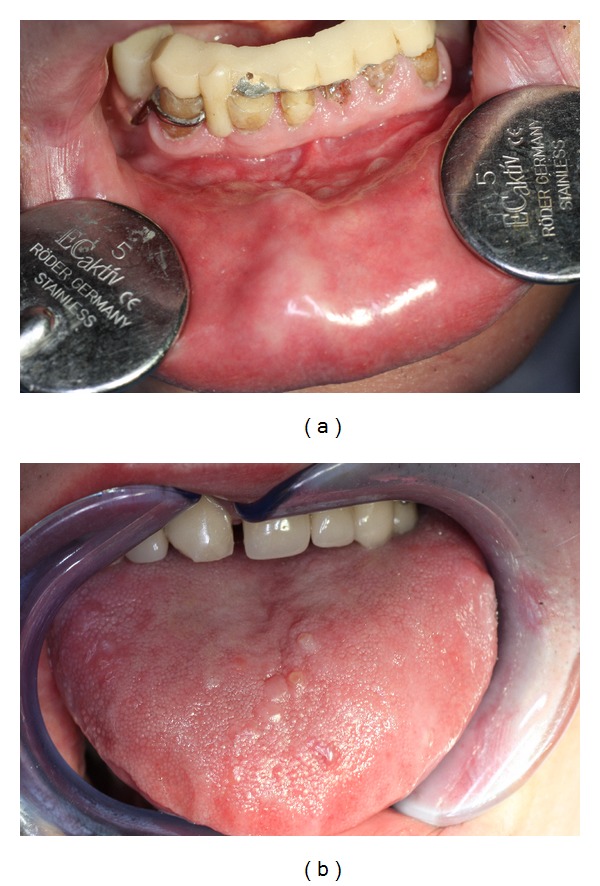
The conditions of oral mucosa in patients with multiple myeloma and amyloidosis (photographs from the collection of the Department of Oral Medicine and Periodontal Diseases, Medical University of Warsaw). (a) Hyperplasia in the mucosa of the lower lip and oral cavity atrium. (b) Exophytic nodular changes characteristic for MM on the dorsal surface of the tongue.

During the examination of oral mucosa, fibrous hypertrophy within upper and lower lip mucosa was felt by palpation, as well as in the arch, in oral cavity atrium, and symmetrically near the corners of the mouth, which according to the patient had been present for several months (Figure 1(a)). It had homogenous structure, irregular shape, and different sizes and did not hurt or bleed. Also slight hardening (scleroderma) of mucosa was observed in the places afflicted by hypertrophy. Microscopic examination of the muscle specimen showed irregularly mixed and hypotrophic fibres with a regular diameter. There were no fibres with central nuclei; some nuclei clusters of nuclear clumps type with fasciculi were diagnosed. Enzymatic colouration kept the differentiation of fibres into three metabolic types with a majority of type 1 fibres and hypertrophy of type 2 fibres (clearly smaller diameter). This allowed diagnosing noncharacteristic changes suggesting myotonic dystrophy type 2 and thus confirmed assumptions concerning simultaneous occurrence of AMD. Characteristic symptoms which appeared before MM allowed diagnosing primary AMD and MM.

Oil solution of vitamin E was prescribed to be applied on the affected oral mucosa 3 times per day from 5 to 7 drops to be spread by the tongue. Spicy, hot food was excluded from the menu. The patient was advised to wash the mouth with lukewarm mallow, flaxseed, and chamomile infusions 3 times a day, after meals.

After one year of treatment for MM, the condition of oral mucosa was worse. Numerous new lesions appeared also on the tongue mucosa, on the dorsal surface, and on its edges (Figure 1(b)). Lesions on the lips and in the cheek mucosa expanded. The patient reported the difficulty to open the mouth wide, to eat, and to use mobile denture for the lower alveolar ridge. His speech was less clear, and he had mobility and manual dexterity problems. Due to the patient's condition, the examination was limited to defining the plaque index (PI) as it is a noninvasive procedure and the index amounted to

- 32% during examination I;
- 35% after one year during examination II.

During the visits the patient was twice instructed in detail how to take care of the mouth hygiene including how to brush his teeth in an atraumatic way. He was advised to use a “soft” toothbrush. It was even suggested that he may use a postoperative toothbrush as it has very delicate bristles of 0.18 mm diameter that can potentially minimize iatrogenic lesions caused by the patient himself. The patient followed these instructions in daily prophylaxis in the hospital and at home. He was also advised to use preventively aseptic and antiseptic preparations in daily mouth hygiene (0.12% chlorhexidine-gluconate solution) as well as alleviative and protective preparations (lukewarm herbal infusions made of mallow, flaxseed, and chamomile). It was also recommended to use vitamin preparations during the therapy, applied directly by the patient on the mucosa of cheeks and fundum of oral cavity and of the tongue (aqueous solution of vitamin A and oil solution of vitamin E were applied on the dorsal surface of the tongue, 5–7 drops to be spread on the surface of oral mucosa), as well as oral supplementation with vitamins: B1, B2, B6, and PP.

## 3. Discussion 

The described case stresses the necessity for various speciality physicians to cooperate, not only for a haematologist and a periodontist, but also for other highly specialised representatives of different fields of medicine such as cardiologists, internists, dermatologists, gastrologists, orthopaedists, nephrologists, radiotherapists, psychooncologists, and physiotherapists. Taking into account growing problems of the bone structure, the patient's orthostatic hypotension, the infection in periodontal tissues will become more severe. This is shown by the plaque index (PI) measurements carried out for one year only. We are thus left with the problem of a complex therapy of oral mucosa and tongue lesions which are pathognomonic for the course of the diagnosed AMD and MM. The authors would like to stress on three issues:

- lesions in the oral cavity during the course of amyloidosis,
- mucositis after bone marrow transplant in MM,
- osteonecrosis of the jaws caused by treatment with bisphosphonates among patients with bone marrow metaplasia.

In the given case, over a span of 5 years, the lesions characteristic for AMD manifested themselves.

It should be expected that the incidence will grow as the average population lifespan is extending. Statistics show that approximately half of cases are diagnosed up to the age of 65 like in the case of this patient. Also the number of cases among people below the age of 40 has been growing. Thus it seems natural that more and more patients suffering from AMD and/or MM will expect from the treating team a therapy that not only alleviates symptoms but also makes recovery realistic. The autologous stem cell transplantation is supposed to allow safe administration of big doses of chemotherapy, thus increasing the chances of recovery. During pancytopenia, after the transplant, patients often suffer from oral mucositis and require periodic administering of narcotic analgesics as well as parenteral nutrition. Mucositis is very painful and during its course a bacterial, viral, or fungal superinfection of the oral mucosa often occurs. Even though the multiple myeloma remains to a great extent an incurable disease, still the introduction of new medication (first thalidomide, then bortezomib and lenalidomide, and recently carfilzomib and pomalidomid) has significantly changed the course of the disease. During the last 15 years a significant progress has been reached in the treatment of this disease. This progress was caused by many factors, including the development of basic knowledge and intensive analysis of new molecules in preclinical trials. This progress extended the average life of patients from mean 2-3 years at the end of the last decade up to 10 years nowadays [9–11, 16].

Osteonecrosis of the jaw usually occurs after a long administration of bisphosphonates. This is caused by osteoclasts—bone resorbing cells coming from the bone marrow that assimilate these preparations. Simultaneously their half life time is reduced and modulation of osteoblasts—bone-forming cells—occurs. These osteoblasts are formed with type I collagen and they are deprived of the capacity to migrate whereas their function in the bone creation and reconstruction strictly depends on the function of osteoclasts. When an organism is healthy, thanks to their phagocytic capacity and their original location in the bone marrow, they form microtubules in the bone structure, thus allowing bone-forming cells to move with blood and by this way the reconstruction process begins. Their long-time impairment leads to osteonecrosis. Bisphosphonates have been accepted in the treatment of hypercalcemia which occurs during MM as they significantly improve the quality of patients' life reducing pain and bone complications. Osteonecrosis is more frequent among MM patients that have been treated with zoledronic acid (Zometa) as compared to the treatment with pamidronic acid (Aredia). Osteonecrosis of the jaws can also occur before, if factors conducive thereto are present, that is, surgery conducted within the oral cavity such as tooth extraction or inflammation as well as poor oral hygiene. Osteonecrosis means an exposed mandibular or maxillary bone visible during a dental procedure, on X-ray photographs, or showing naturally, without any signs of healing after six weeks of treatment and dental care. Before the treatment with bisphosphonates is introduced, it is necessary to carry out a dental examination and sanitation of oral cavity. Dentists should carefully remove every focus of infection within oral cavity before bisphosphonates are introduced or during the first few weeks since their introduction. According to American standards gathered in Mayo Clinic, bisphosphonates should be administered to MM patients for one year and a half up to two years and later depending on the patient's condition. During the therapy, tooth extraction and inserting tooth implants should not be carried out and other dental procedures should be done with great care. Extensive surgery should be avoided. If osteonecrosis of the jaws occurs during treatment with bisphosphonates any dental procedure may worsen the condition [17–23].

Taking into account a very limited scope of the therapy during the initial phase, lack of activities during the corrective phase, and recommendations for the maintenance phase, the authors hope for at least a partial elimination of the potential source of mucositis and of the infection originating in periodontal tissue that may play an auxiliary role during the therapy of the basic disease, improving the comfort and the quality of the patient's life [24, 25].

This paper points out that a comprehensive therapy is necessary as well as an interdisciplinary approach to the treatment of AMD and/or MM patient.

## 4. Clinical Relevance

The presented case of a 62-year-old man with MM documents the changes of oral mucosa and periodontal tissues due to MM itself (amyloidosis), as well as the applied treatment (bisphosphonates). Often such changes do not become the focus of attention although the periodontal state may impact the quality of life. The periodontal consultancy should be therefore the essential part of MM treatment. The standard periodontal medication should be introduced. The above subject of periodontal status in MM has been rarely discussed in medical literature.
